# Systemic virus infection results in CD8 T cell recruitment to the retina in the absence of local virus infection

**DOI:** 10.3389/fimmu.2023.1221511

**Published:** 2023-08-18

**Authors:** Egle Paskeviciute, Mei Chen, Heping Xu, Bent Honoré, Henrik Vorum, Torben Lykke Sørensen, Jan Pravsgaard Christensen, Allan Randrup Thomsen, Mogens Holst Nissen, Maria Abildgaard Steffensen

**Affiliations:** ^1^ Department of Immunology and Microbiology, University of Copenhagen, Copenhagen, Denmark; ^2^ Wellcome-Wolfson Institute for Experimental Medicine, School of Medicine, Dentistry & Biomedical Science, Queens University of Belfast, Belfast, Ireland; ^3^ Department of Biomedicine, Aarhus University, Aarhus, Denmark; ^4^ Department of Clinical Medicine, Aalborg University, Aalborg, Denmark; ^5^ Department of Ophthalmology, Aalborg University Hospital, Aalborg, Denmark; ^6^ Department of Ophthalmology, Zealand University Hospital, Roskilde, Denmark; ^7^ Faculty of Health Sciences, University of Copenhagen, Copenhagen, Denmark

**Keywords:** retina, lymphocytic choriomeningitis virus (LCMV), CD8 T cell, retinal pigment epithelial cell, CXCR6, CXCL16, immune surveillance

## Abstract

During recent years, evidence has emerged that immune privileged sites such as the CNS and the retina may be more integrated in the systemic response to infection than was previously believed. In line with this, it was recently shown that a systemic acute virus infection leads to infiltration of CD8 T cells in the brains of immunocompetent mice. In this study, we extend these findings to the neurological tissue of the eye, namely the retina. We show that an acute systemic virus infection in mice leads to a transient CD8 T cell infiltration in the retina that is not directed by virus infection inside the retina. CD8 T cells were found throughout the retinal tissue, and had a high expression of CXCR6 and CXCR3, as also reported for tissue residing CD8 T cells in the lung and liver. We also show that the pigment epithelium lining the retina expresses CXCL16 (the ligand for CXCR6) similar to epithelial cells of the lung. Thus, our results suggest that the retina undergoes immune surveillance during a systemic infection, and that this surveillance appears to be directed by mechanisms similar to those described for non-privileged tissues.

## Introduction

Traditionally, the retina and CNS are viewed as separate entities that are isolated from the remaining organ systems when it comes to the response to an infection. This concept is called immune privilege and is believed to be in state to protect the fragile neural tissue from damage caused by a full-blown inflammatory response. Both physical barriers as well as anti-inflammatory components within the tissue itself enforce immune privilege (1–3). However, recent results have demonstrated that both the retina and the brain may be more integrated in the systemic response to infection than was previously believed (4–6). Urban et al., 2020 showed that CD8 T cells enter the brain tissue after both systemic and intranasal infections, while Voigt et al., 2018 demonstrated CD8 T cells in the retina after systemic chronic CMV infection that causes broad ocular infection. However, it is unknown whether CD8 T cells also enter and remain in the retina during an acute systemic virus infection, i.e. a setting where the immune response rapidly clears the virus from the organism and there is no chronicity. LCMV Armstrong (LCMV Arm) is a well-known murine pathogen that induces an acute and quickly resolved infection in the host. Clearance of virus is dependent on a potent CD8 T cell response. It is unknown whether LCMV Arm can reach and actively infect cells of the retina via the intravenous route, but previous studies have shown that only minute amounts of virus may be found in the CNS, an organ that is immunologically similar to the retina (7, 8). It has previously been shown that mRNA encoding CD8 is enriched in the RPE and choroid (RPE/c) after i.v. LCMV Arm infection (9), but it is unknown whether CD8 T cells actually leave the vessels of the choroid and enter the retinal tissue itself. The retina is at the center of the visual process. It contains both photoreceptors that sense light and neurons that integrate and transform signals from the photoreceptors into images that are sent to the brain via the visual nerve. Inflammatory damage in the retina can therefore be detrimental as photoreceptors and neurons do not possess the ability to regenerate. Depending on the detailed location of retinal damage, the outcome may be different kinds of visual disturbances (10–12). Knowledge on how the immune response interacts with the retina during a relatively harmless viral infection is relevant to understand the dynamics between the response to infection in systemic organs versus that in the privileged retina, and thus for understanding the pathogenesis of disease in the retina. With background in the knowledge that has emerged in recent years, the central question for this study was whether immune surveillance occurs in the retina as it does in other organs. Studies on immune surveillance in organs such as the liver and lungs have shown that the chemokine receptor CXCR6 and its ligand CXCL16 are important for migration of CD8 T cells in the tissue during an infection in mice. CXCL16 expressed by epithelial cells in the liver and lungs was shown to be responsible for creating a chemotactic gradient that directed CD8 T cell localization within the tissue (13–15). Retinal pigment epithelial (RPE) cells are epithelial cells that line the outermost part of the retina and they are extremely important for the homeostasis and proper function of the photoreceptor layer. Further, they constitute what is known as the outer blood retinal barrier by strictly controlling entry of nutrients into the retina. Previous studies have shown that RPE cells can produce a number of chemokines in response to inflammatory stimulus *in vitro*, and expression of chemokine genes in the RPE/c has also been shown in response to infection *in vivo* (9, 16). We therefore speculated whether RPE cells might play a role in CD8 T cell trafficking within the retinal tissue.

By using the LCMV Arm model, we investigated CD8 T cell entry into the retina after a transient systemic infection, as well as the location and phenotype of cells within the retinal tissue. Based on the phenotype of CD8 T cells within the retina, we explored expression of chemokine ligands in RPE cells both *in vitro* and *in vivo*. We found CD8 T cells in the space between RPE cells and the choroidal vessels, and in several layers of the neural retina, including the inner and outer plexiform layer, as well as the photoreceptor outer segment layer. We also found that CXCR6 was expressed by the majority of CD8 T cells in all compartments of the retina and choroid, and that CXCR3 was differentially expressed on CD8 T cells within the neuroretina. We found that RPE cells constitutively express CXCL16 in the membrane, and that the chemokine was secreted during inflammatory conditions. Based on these findings, we speculate that a CXCR6-CXCL16 axis may exit between CD8 T cells and RPE cells as part of directing immune surveillance of the retinal tissue, similar to what has been reported for the lung and liver.

## Materials and methods

### Mice and infection

Female C57BL/6 wildtype mice were purchased from Taconic Biosciences. All mice included in this study were 8-12 weeks old at start of experiments, and they were housed in individually ventilated cages under specific pathogen free conditions at the animal facility at the University of Copenhagen. Mice were allowed to rest for at least 1 week before they were included in experiments. LCMV Arm 53b was administered intravenously in a concentration of 10^4^ PFU (plaque forming units) in 300 µl PBS for systemic infection. Titer of virus stock was determined in a focus-forming assay as previously described (17). The animal experiments described in the current study were approved by the Danish Animal Experiments Inspectorate with license number 2020-15-0201-00586. Mice were euthanized by cervical dislocation in a swift manner by trained staff in accordance with the license given by the Danish Animal Experiments Inspectorate.

### Harvest of ocular tissue and dissection

Eyes were harvested by cutting the optic nerve immediately after cervical dislocation and kept on ice during the whole dissection procedure. For flow cytometry analysis, eyes were dissected under a microscope as previously described (18). First, the remaining muscles and connective tissues were removed with scissors. Then, the eye was held with tweezers and a round cut below the lens was made to cut the ciliary body and remove the lens. The neural retina was harvested first, and RPE/c was scrapped off from the eye cup using an 18-gauge needle. Neural retinas and RPE/c were collected separately in cold Hanks Balanced Salt Solution (HBSS). RPE/c and neuroretinas from the eyes of 1 mouse were pooled together. For histology, whole eyes from 1 mouse were immersed in a biopsy container with a 4% formaldehyde solution (BiopSafe^®^, cat. 31.78.200-03 DK)).

### Detection of viral RNA in retina and spleen

RPE/c, neuroretina and spleen were harvested in RNA*later* (ThermoFisher Scientific, cat. AM7020) and stored at -80 degrees. Total RNA was extracted from homogenized tissue by the NucleoSpin RNA mini kit (AH Diagnostics, cat. 740955) according to the manufacturer’s protocol.

RNA was reverse transcribed to cDNA by using a First-strand cDNA synthesis kit (ThermoFisher Scientific, cat. K1622) according to the manufacturer’s instructions. The amount of RNA transcribed varied between 0.1-0.5 µg depending on tissue. LCMV RNA (nucleoprotein) was detected by TaqMan qPCR using primers and probes previously described (19). A GAPDH expression assay (ThermoFisher Scientific) was included as a housekeeping control. Expression of LCMV nucleoprotein relative to GAPDH was calculated using the delta delta Ct method. A standard curve with dilutions of viral RNA purified from the virus stock was included in all experiments to establish the limit of detection and ensure the overall quality of the analysis. According to the standard curve, a viral RNA dilution corresponding to 0.5 Pfu will give a Ct of approximately 38, i.e. the LCMV NP qPCR will detect minute amounts of virus if present in the tissue (**Supplementary Figure 1C**). Further, we know from an ongoing study that it is possible to detect LCMV NP in RPE/c and neuroretina with the primers and probe used in the current study when a different type of infection is established (manuscript in preparation).

### Histology

Paraffin-embedded eyes were cut in sections of 4 µm and dewaxed with xylene, alcohol and tap water.

For hematoxylin/eosin (HE) staining, eye sections were incubated in Mayer’s Hemalum solution (Merck, cat. 1092490500) for 5 minutes, washed for 5 minutes in tap water, followed by incubation in Eosin Y (Merck, cat. HT110132) for 5 minutes. Finally, sections were rinsed in 70% ethanol, 96% ethanol and 99% ethanol.

For immunohistochemistry, antigens were retrieved by placing the tissue sections in a TEG buffer (10 mM Tris, 0.5 mM EGTA, pH9), followed by boiling in a microwave oven for 15 min. After this, samples were pre-incubated in 2% BSA for 10 minutes before incubation with primary antibody (α-CD8 or α-CXCL16) overnight at 4°C. Sections were then washed in buffer and incubated in biotinylated secondary antibody for 40 minutes. After washing, endogenous peroxidase was blocked in 3% hydrogen peroxide. Then it was incubated with VECTASTAIN ABC reagent (Bionordika, cat. VEC-PK-4000) for 30 min, and the reaction was developed using 3.3-diaminobenzidine (DAB, KEM-EN-TEC, cat. 0215082691-06) for 15 minutes. The procedure was finished by counterstaining with Mayer’s Hemalum solution (Merck, cat. 1092490500) and rinse.

For immunofluorescence, the procedure was similar, but sections were incubated in fluorochrome labeled secondary antibody, followed by blocking and rinse.

The staining procedures were performed at the Department of Biomedical Sciences, University of Copenhagen.

### Flowcytometry

To enable distinction between cells residing in capillaries and actual tissue residing cells, mice were administered 3 µg PE-CF594 labeled α-CD45 intravenously 10 minutes prior to removal of eyes and spleens. Singe-cell suspension of spleens was generated by pressing the organ through a 70µm cell strainer (Merck, cat. CLS431751) followed by centrifugation in HBSS. Eyes were dissected and single cell suspensions were generated by digestion with Liberase (Merck, cat. 5401020001). RPE/c was digested in a 0.025% Liberase solution, while neuretinas were digested in a 0.0075% Liberase solution. Both tissues were digested for 20 minutes at 37°C in the presence of 5 µg/ml DNase (Merck, cat. D4263). To obtain single-cell suspensions, digested tissue was passed through a 70 µm cell strainer followed by centrifugation. Cells were resuspended in HBSS and all cells from 1 mouse were transferred to a single well in a 96 well U-bottom plate. For surface staining, the cells were incubated with 50 μl FACS medium (PBS, 1% BSA, 0.1% NaN3) containing α-CD16/32 (1:100) to block F_c_ receptors for 10 minutes at 4°C. After this, cells were incubated for 20 minutes at 4°C in the dark with 50 μl brilliant violet staining buffer (BD biosciences, cat. 563794) containing conjugated antibodies (1:100) for the t cell-surface markers CD45, CD8, LFA-1, CXCR6, CXCR3 and CD69. After staining and fixation, cells were stored at 4°C until flowcytometric analysis the following day.

### Antibodies

The following fluorochrome-conjugated anti-mouse monoclonal antibodies were used for flow cytometry: α-CD16/32 (cat. 553141), α-CD8 (APC-Cy7, cat. 561967), α -CD45.2 (PerCP-Cy5.5, cat. 561096, PE-CF594, cat. 565390), α-LFA-1 (BV605, cat. 740340), α-CXCR6 (APC, cat. 151105), α-CXCR3 (BV650, cat. 740630), α -CD69 (PE, cat. 561932). Antibodies were purchased from BD Bioscience with the exception of α-CXCR6 (Nordic Biosite). The following non-conjugated antibodies were used for immunohistochemistry and immunofluorescence: α-CD8 (Nordic Biosite, cat. CST-98941S), α-CXCL16 (Nordic Biosite, cat. bs-1441R), biotinylated goat anti-rabbit (BioNordika, cat. VEC-BA-1000-1.5), and Alexa 488 goat anti-rabbit (Abcam, cat. 150081).

### Immunoassays

The B6-RPE07 cell line is a spontaneously arisen primary murine RPE cell line isolated from a 12 weeks old female C57BL/6 mouse. The cells display a cobblestone morphology and have previously been shown to maintain barrier function when grown on transwell inserts (20). B6-RPE07 cells were routinely cultured in DMEM high glucose medium (Sigma-Aldrich, cat. D6429) containing 10% FBS (Merck, cat. 35079-CV). For investigation of chemokine expression, cells were seeded on transwell inserts (Greiner Bio-One, cat 662641) in a 12-well plate at a concentration of 10,000 cells/cm^2^. Transwell inserts allow for polarization of epithelial cells and enable differentiation of substances secreted from the apical and basolateral side. When cells reached confluence, they were allowed to stabilize in a serum free medium (X-vivo, BioNordika, cat. 02-060 F) for 24 hours before stimulation with IFN-γ (RnD Systems, cat. 485-MI-100). Stabilized cell layers with cobblestone morphology were stimulated with 100 ng/ml IFN-γ for 48 hours before cells and supernatants were harvested and stored at -80°C. Supernatants were harvested carefully using a pipette and after transfer to eppendorf tubes, the samples were centrifuged at 1500 rpm for 10 minutes at 4°C. Cell pellets were lyzed using cell lysis buffer II (ThermoFisher, cat. FNN0021) according to the manufacturer’s instructions. In brief, cell pellets were resuspended in lysis buffer at a concentration of 10^8^ cells/ml and incubated for 30 minutes on ice with vortexing at 10-minute intervals. Lysates were cleared by centrifugation at 13,000 rpm for 10 minutes at 4°C and stored at -80°C. Tissue lysates were prepared by first dissecting the mouse eye to isolate the RPE/c and then transfer the tissue to cell lysis buffer II followed by homogenization. Lysates were cleared by centrifugation at 13,000 rpm for 10 minutes at 4°C and stored at -80°C. CXCL16 in lysates and supernatants was assessed by an anti-mouse CXCL16 ELISA kit according to the manufacturer’s instructions (Thermofisher, cat. EMCXCL16). CXCL16 concentrations were normalized to protein content according to a standard bicinchoninic acid (BCA) protein quantification assay. Cytokines in serum were assessed by a multiplex immunoassay (V-PLEX Proinflammatory Panel 1 (mouse) kit) according to the manufacturer´s instructions (Meso Scale Diagnostics, cat. K15048D-1).

### Statistical evaluation

GraphPad Prism software (version 9) was used for statistical analyses. Quantitative results were compared using a nonparametric Mann-Whitney U-test and a p-value of <0.05 was considered evidence of a statistically significant difference. P <0.05: *, p<0.01: **, p<0.001: ***, p<0.0001: ****.

## Results

### Systemic infection with LCMV Arm does not result in active infection of the retina, and there are no obvious signs of inflammation in the retinal tissue

**Figure 1:**
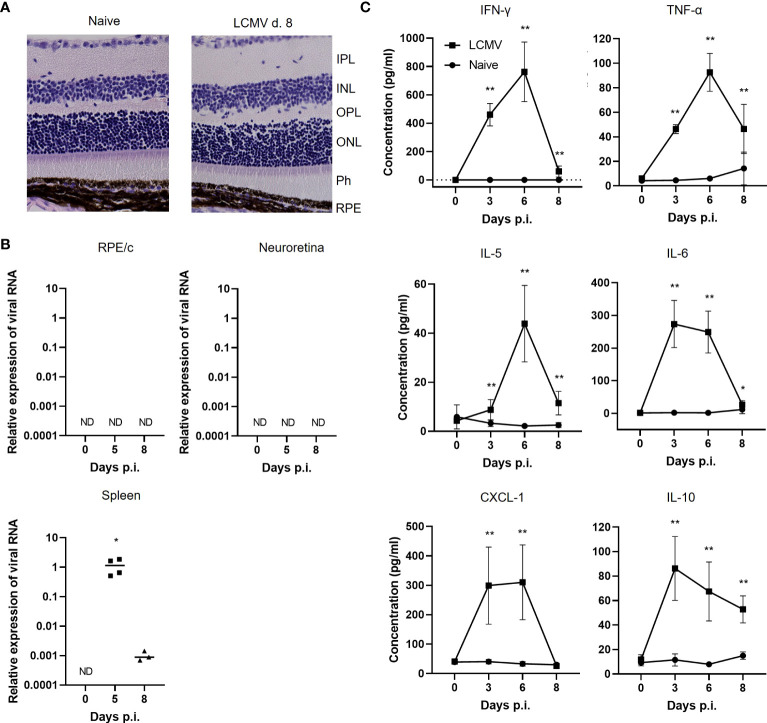
Systemic LCMV infection does not result in active infection of the retina. **(A)** HE staining of retinas from naïve or infected mice day 8. **(B)** Relative expression of viral RNA/GAPDH RNA in RPE/C, neuroretina and spleen in naïve and infected mice day 5 and 8.**(C)** Cytokine levels in serum from naïve and infected mice day 3, 6 and 8. Data represents 2-3 independent experiments. A nonparametric Mann-Whitney U-test was used to compare groups, p <0.05: *, p<0.01: **.

In a previous study, we observed upregulation of CD8 RNA in the RPE/c after LCMV Arm infection (9), and in the current study we wanted to elaborate on this finding by investigating the presence of CD8 T cells in both RPE/c and neuroretina. As a starting point, we wanted to establish whether an acute systemic infection with LCMV Arm leads to active infection of the retina and/or structural changes within the layers of the retina such as retinal vessel enlargement and inflammatory cells in the subretinal layer as a sign of active inflammation. To investigate the structural integrity of the retina, we infected mice with LCMV Arm i.v., performed HE staining on days 5, 8 and 30 after infection, and compared these with naïve mice. On day 5 after LCMV Arm infection, viral titers peak in most organs and infectious virus is usually cleared from the system by day 8 (8). Day 30 after infection was included to ensure that there were no late effects of the virus infection in the retina. Histological sections of retinas from infected mice showed normal structure and no obvious inflammatory cell infiltration or vessel enlargement at all time points (**Figure 1A**).

Since retina is an immune privileged site and LCMV is a non-cytopathic virus, virus could be present without any obvious signs of inflammation. We therefore evaluated the presence of virus by a previously described qPCR detecting RNA encoding the LCMV nucleoprotein (19). For detection of viral RNA, we dissected eyes and collected the neuroretina and RPE/c compartments for RNA purification and qPCR. RPE/c and neuroretina represent 2 quite distinct compartments immunologically as the neuroretina is somewhat shielded from the circulation, whereas the RPE cells are in close contact with the large vessels in the choroid. It was therefore plausible that we would find virus in one compartment and not the other. However, we did not detect any viral RNA in any of the retinal compartments on days 5 or 8 after infection, whereas virus could readily be detected in the spleen (**Figure 1B**). Housekeeping gene (GAPDH) expression was similar between the tissues (**Supplementary Figure 1**).

The posterior part of the eye, which includes both the RPE/c and neuroretina, is a heavily vascularized tissue due to high metabolic demands, and serum components could therefore have a significant impact on this tissue. Previous studies have shown that RPE cells can express a number of pro-inflammatory cytokines and chemokines upon stimulation with inflammatory cytokines both *in vitro* and *in vivo* (9, 16, 21). We therefore measured a panel of cytokines in serum from infected animals to investigate which circulating inflammatory mediators that could potentially affect RPE/c and neuroretina during an LCMV infection. As shown previously (9), we transiently found large amounts of IFN-γ in serum from infected animals, and the response peaked on day 6 after infection. Following similar kinetics, we also found large amounts of TNF-α, IL-5, IL-6, CXCL-1 and IL-10 in serum from acutely infected animals (**Figure 1C**). Thus, even though virus is absent from the RPE/c and neuroretina, these tissues could potentially be impacted by the large amounts of cytokines in circulation during an LCMV infection.

### Systemic LCMV Arm infection leads to CD8 T cell surveillance of the retina

**Figure 2:**
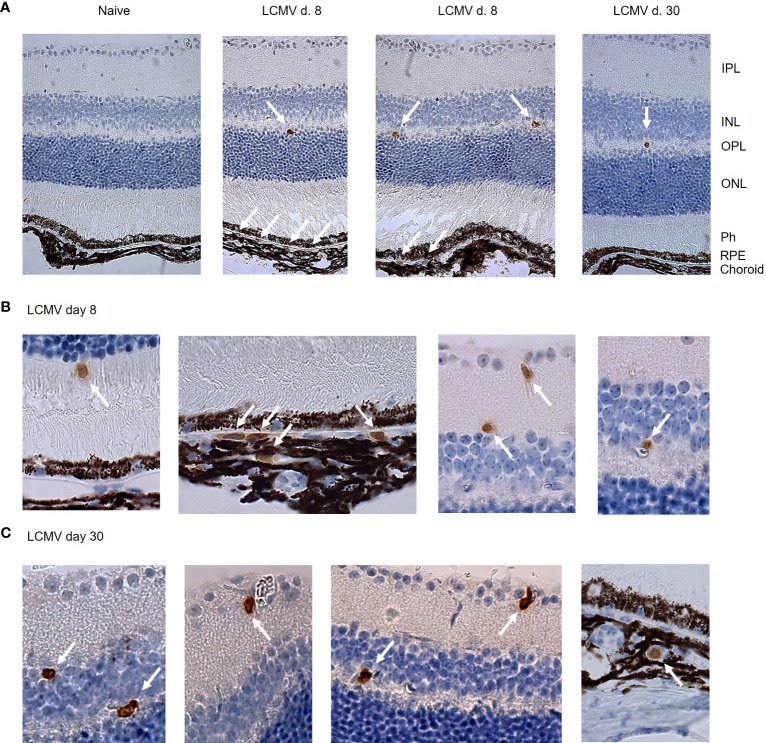
Immunohistochemistry shows CD8 T cells in the RPE/c and neuroretina after systemic LCMV infection. (A) CD8 staining of retinal sections from naïve an infected mice day 8 and 30. (B, C) Close-up of CD8 positive cells in RPE, choroid and neuroretina on day 8 and 30 after infection. Arrows mark CD8 positive cells. Data represents 2 independent experiments.

LCMV Arm elicits a substantial CD8 T cell response that peaks around day 8, and on day 30 after infection, the population of virus-specific CD8 T cells has contracted into a stable memory pool (8). To evaluate CD8 T cell surveillance of the retina, we initially investigated the presence of CD8 T cells in the retina and choroid on days 8 and 30 after infection by immunohistochemistry staining. The choroid is a vascular tissue that is in close contact with the RPE cell layer. CD8 T cells that leave the choroidal vessels could therefore potentially interact with RPE cells and affect the neuroretina indirectly, while CD8 T cells found within the neuroretina could affect the retinal tissue more directly. On day 8 after LCMV infection, CD8 T cells were found in both the choroid and retina (**Figures 2A, B**), while at the memory stage of the CD8 T cell response (day 30), we found only few CD8 T cells still residing in retina and choroid. (**Figures 2A**,C). Within neural retina, CD8 T cells resided in the inner and outer plexiform layers (IPL and OPL), where synapses between the neural cells of the retina are located (**Figures 2B, C**). We also found CD8 T cells in the avascular photoreceptor layer (**Figure 2B**). We did not find CD8 T cells in retinas from naïve mice (**Figure 1A**).

### Intravascular staining reveals 2 distinct populations of vessel and tissue residing CD8 T cells in retina

**Figure 3:**
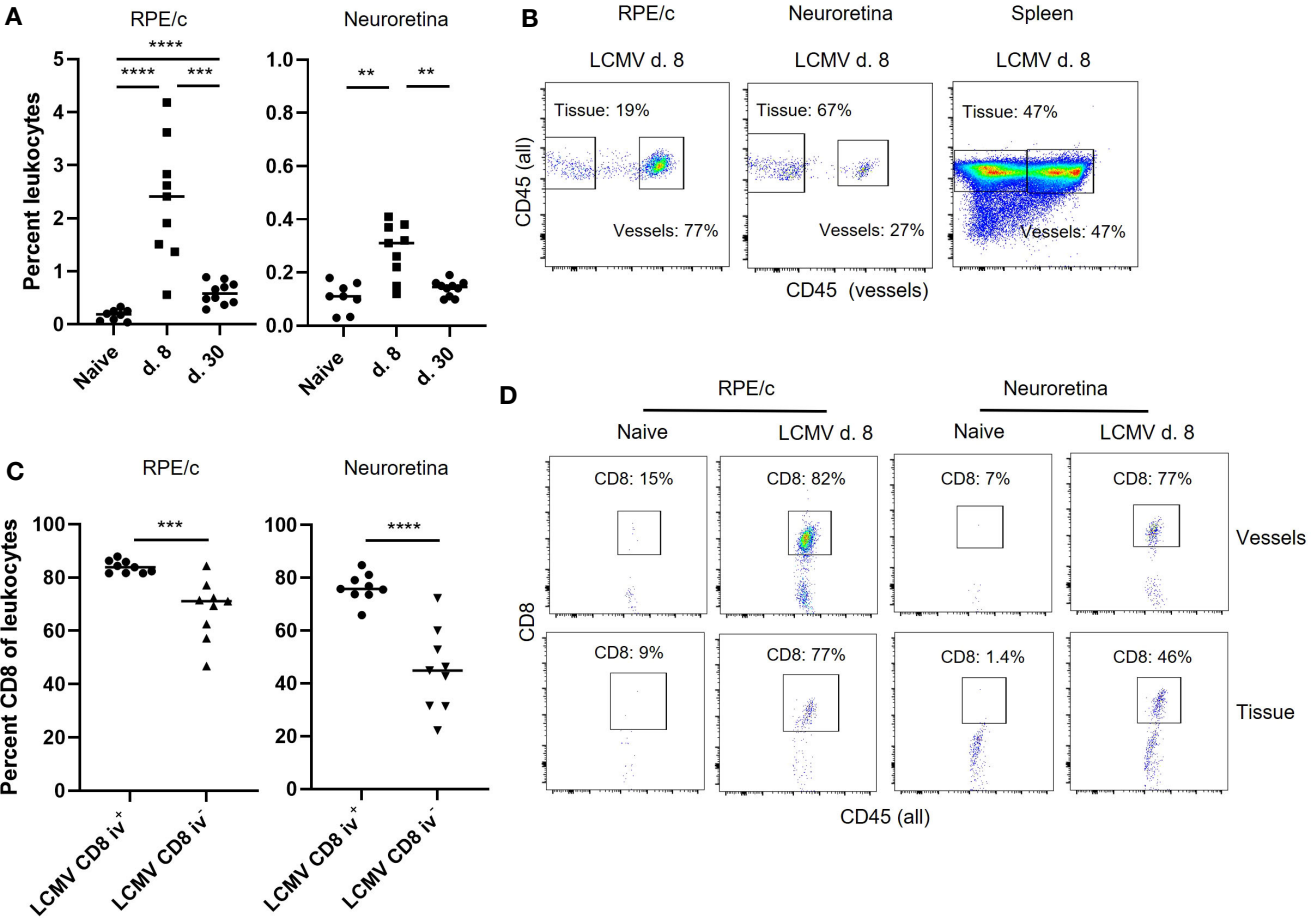
Intravascular staining reveals the presence of vessel and tissue residing CD8 T cells in retina. **(A)** Leukocytes (CD45+) infiltrating the RPE/c and neuroretina on day 8 after infection. **(B)** anti-CD45 (PE-CF594) antibody was injected into mice before euthanization, and single cell suspensions of RPE/c, neuroretina and spleen were stained with CD45 (PerCP-Cy5.5) to separate the leukocytes population into those residing in the vessels (double positive) and those residing in the tissue (single positive). Dot plots represent mice infected with LCMV 8 days earlier. **(C)** Percent CD8 T cells found in RPE/c and neuroretina from infected mice day 8. **(D)** Representative dot plots showing CD8 T cells in the tissue and vessels of the neuroretina and RPE/c from naïve and infected mice (d. 8). Data represents 3 independent experiments. A non-parametric Mann-Whitney U-test was used to compare groups, p < 0.01: **, p < 0.001: ***, p < 0.0001: ****.

CD8 T cell infiltration can be driven both by the presence of local tissue inflammation and by signals derived from parenchymal cells responding to circulating inflammatory mediators. As we could not detect viral RNA within the retina (**Figure 1B**), we assumed that CD8 T cell influx was driven by general mechanisms also present in other tissues of the body during infection. We therefore proceeded to investigate the phenotype of CD8 T cells found in RPE/c and neural retina compartments after systemic infection with a focus on markers previously shown to impact CD8 T cell homing (13–15). To separate tissue residing cells from intravascular cells, we used a previously established method of intravascular staining to label leukocytes in circulation (22), followed by dissection of eyes and staining of CD8 T cells found in the RPE/c and neural retina compartments. We found a population of CD45^+^ cells (leukocytes) in both compartments on day 8 and 30 after infection. In line with our immunohistochemistry results, the leukocyte population found on day 30 after infection was very small and similar to that in naïve controls, indicating that the majority of infiltrating cells had left both compartments at this time point (**Figure 3A**). Separation of leukocytes into iv+ and iv- populations revealed that the majority of leukocytes found in the RPE/c compartment at day 8 were located in the vessels, whereas the majority of leukocytes found in the neural retina resided within the tissue. As expected, approximately 50% of leukocytes in spleens from naïve mice were stained with the iv antibody, validating the intravascular staining procedure (**Figure 3B**). CD8 T cells were found in both RPE/c and neural retina from infected animals, and they generally represented more than half of the leukocyte population recovered from these sites on day 8 after infection (**Figure 3C**). A few CD8 T cells could be found in the RPE/c from naïve animals, but we did not detect any CD8 T cells in neural retinas from naïve controls (**Figure 3D**).

**Figure 4:**
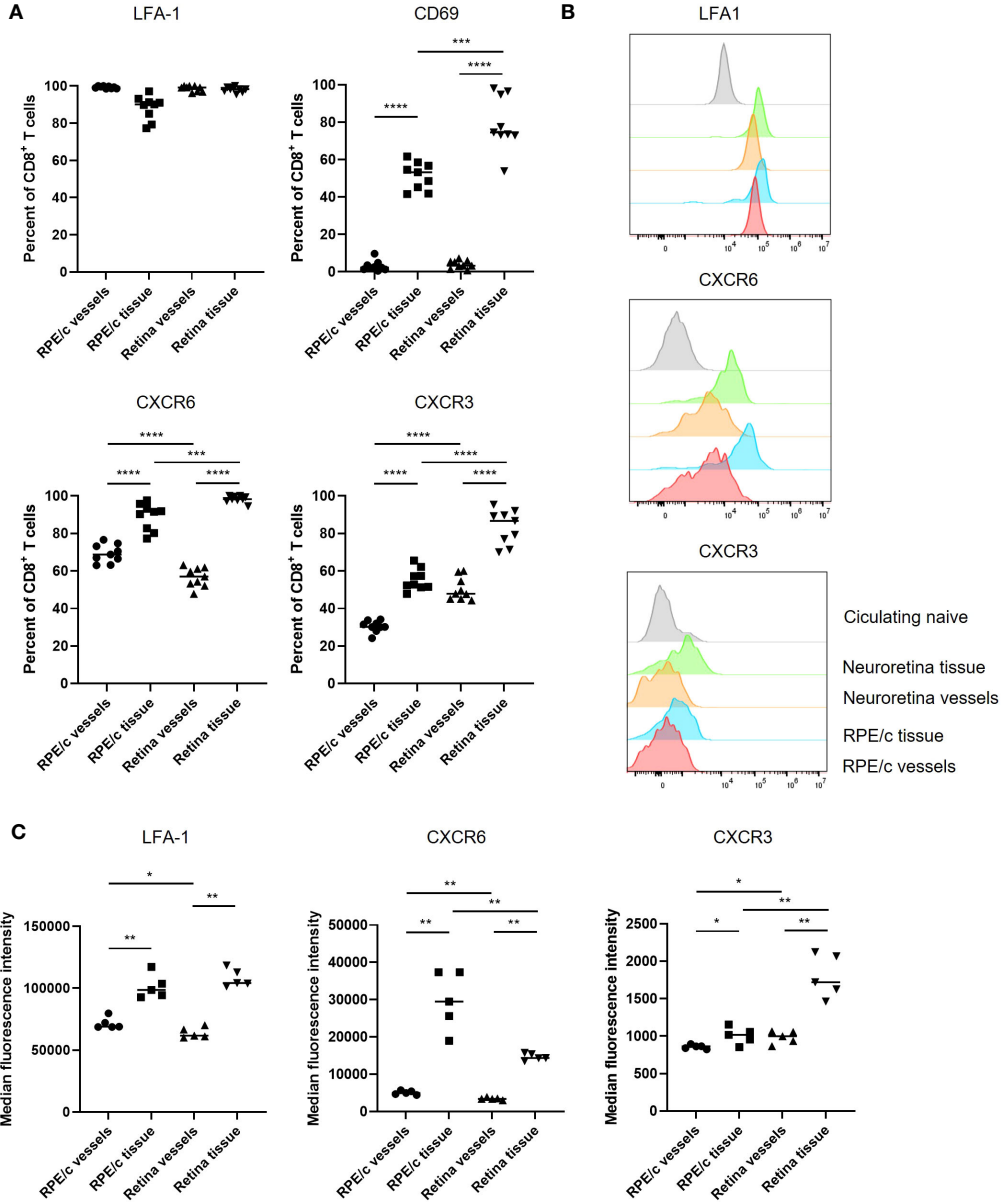
CXCR6 and CXCR3 expression is enriched on CD8 T cells residing in the tissue. **(A)** Expression of phenotypic markers by CD8 T cells infiltrating the RPE/c and neuroretina compartments on day 8 after infection. **(B)** Representative histograms showing expression levels of phenotypic markers on day 8 after infection compared to expression level in naïve circulating CD8 T cells (grey). **(C)** Cumulative data showing expression levels (MFI) of phenotypic markers on CD8 T cells infiltrating the RPE/c and neuroretina compartments on day 8 after infection Data represents 3 independent experiments. A non-parametric Mann-Whitney U-test was used to compare groups, p<0.05: *, p<0.01: **, p<0.001: ***, p<0.0001: ****.

We proceeded to characterize CD8 T cells present in RPE/c and neural retinas on day 8 after infection using markers typically responsible for endothelial adhesion (LFA-1), retention in tissues (CD69) and migration/positioning (CXCR6, CXCR3). The CD8 T cells found within the vessels of the RPE/c and neural retina compartments (iv^+^) were similar in phenotype, i.e. 50-70% of the cells expressed CXCR6, they all expressed LFA1, but they did not express CD69. When CD8 T cells had left the vessels (iv^-^), CXCR6 expression was enriched to 90-100% of the population, and CD69 was upregulated. In neural retina, the majority of CD8 T cells were positive for CD69 while approximately 50% of cells in the RPE/c iv^-^ compartment expressed CD69. CXCR3 was expressed on approximately 30% of CD8 T cells in the vessels of the RPE/c compartment (RPE/c iv^+^), and was enriched to 50-60% of the CD8 T cells in RPE/c iv^-^ compartment. In the vessels of neural retina, approximately 50% of CD8 T cells expressed CXCR3, while within the retinal tissue, the vast majority (70-95%) expressed CXCR3 (**Figure 4A**). Further, expression levels of this chemokine receptor was also higher on CD8 T cells within the neural retina when compared to the other compartments (**Figures 4B, C**). We also observed changes in expression levels of CXCR6 and LFA1 depending on compartment such that expression level of both markers increased from circulating to tissue residing cells. These observations are in line with previous studies on CD8 T cells infiltrating the lungs and the liver (13, 23) (**Figures 4B, C**). Interestingly, we consistently observed a higher expression level of CXCR6 on CD8 T cells within the RPE/c iv^-^ compartment when compared to all other compartments. Gating strategy for flow cytometry can be seen in **Supplementary Figure 2**.

### CXCL16 expression in RPE cells

**Figure 5:**
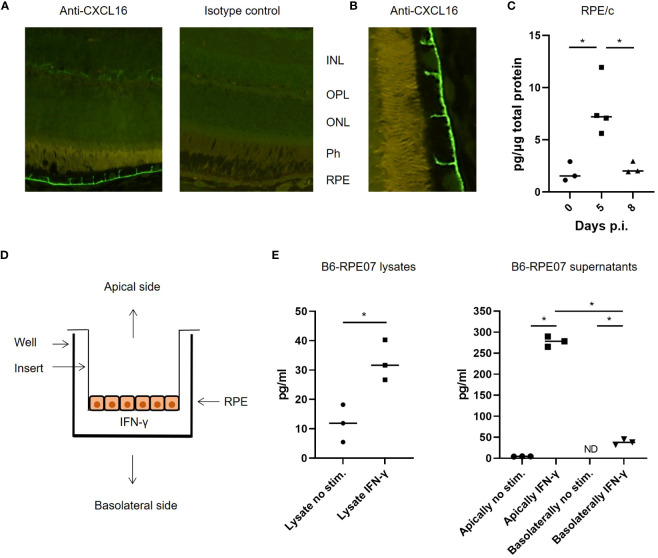
CXCL16 expression by RPE cells. **(A)** representative immunofluorescence staining for CXCL16 expression in retinal sections, no difference was observed between day 0, 5 and 8 after infection with this method. **(B)** Close-up showing CXCL16 staining of the apical membrane of RPE cells **(C)** CXCL16 protein in RPE/c lysates from naïve and infected mice day 5 and 8. **(D)** Setup for cell stimulation assays **(E)** CXCL16 protein in lysates and supernatants from IFN-γ stimulated B6-RPE07 cells cultured on inserts. Data represents 2-3 independent experiments. A nonparametric Mann-Whitney U-test was used to compare groups, p < 0.05: *.

A recent study showed that lung epithelial cells express the ligand for CXCR6 (CXCL16), and that CXCR6-CXCL16 interaction drives CD8 T cell localization within the lungs after an influenza infection (13). Since the majority of CD8 T cells found in the RPE/c and retina after LCMV infection express CXCR6, we wanted to investigate whether RPE cells could also express CXCL16 and thereby play a role in the migration of CD8 T cells in the retina. We initially performed immunofluorescence staining of retina sections from naïve and infected mice with an antibody specific for CXCL16, and found that RPE cells expressed CXCL16 in their membrane in both naïve and infected mice (**Figures 5A, B**). Expression was limited to the basolateral membrane, no staining was seen on the apical side of the cells, and fluorescence intensity was similar between infected mice 5 and 8 days post infection and naïve controls. The specificity of CXCL16 staining was confirmed by staining sections with an isotype control antibody as well as with secondary antibody alone (**Figure 5A** and data not shown). Further, the specificity of the antibody was previously tested in CXCL16 knockout mice (13). We next investigated whether ELISA could confirm CXCL16 expression in tissue lysates from LCMV infected animals. As evident from **Figure 5C**, CXCL16 protein was detected in RPE/c lysates with ELISA, and we detected an increase in protein levels on day 5 after infection. The increase in CXCL16 protein levels in the RPE/c could be caused by 3 factors: Increased CXCL16 levels in the choroidal vessels (unrelated to RPE expression), increased membrane expression not detectable by immunohistochemistry or increased secretion of CXCL16 from the RPE cells.

To confirm that CXCL16 was constitutively expressed in the membrane of RPE cells and to investigate whether the chemokine is secreted by RPE cells during inflammatory conditions, we took advantage of a spontaneously arisen primary murine RPE cell line described previously (20). Since the RPE/c and retina were not actively infected, we assumed that any effect of the infection on the RPE cells would arise from inflammatory mediators in circulation. It has previously been shown that chemokine expression by the RPE/c in response to LCMV infection is primarily driven by IFN-γ (9), and we therefore proceeded to investigate the secretion of CXCL16 from primary murine RPE cells stimulated with IFN-γ. The RPE cells were seeded in a trans well insert to enable separation of CXCL16 secreted from the basolateral and apical side of the RPE cells (**Figure 5D**). Cells were stimulated for 48 hours, and protein expression was measured in supernatants and cell lysates by ELISA. As evident from **Figure 5E**, constitutive expression of CXCL16 in RPE cells was confirmed. We also observed an increase in CXCL16 expression in cell lysates after stimulation. Minute amounts of CXCL16 were secreted from unstimulated RPE cells whereas secretion increased from both the basolateral and apical side after stimulation. The vast majority of CXCL16 was secreted from the apical side of the RPE cells (towards the retina), which would be consistent with a role in directing the migration of CXCR6^+^ CD8 T cells within the retinal tissue.

## Discussion

The presence of CD8 T cells in the retina and CNS was previously viewed as a sign of pathology. However, recent discoveries have indicated that neurological tissue may be more integrated with the general immune response than previously believed, and CD8 T cells have been found in both the retina and CNS after systemic infections even without the presence of local virus infection (4, 5). Thus, there is emerging evidence that neurological tissues are not isolated from the circulation during a systemic immune response.

In the current study, we investigated CD8 T cell infiltration of the retina in the context of an acute and easily resolved systemic infection with LCMV Arm where no virus is present in the retina, but where local chemokine production is induced in response to increased systemic IFN-γ levels (9). We found that CD8 T cells leave the vessels of the retina and enter the retinal tissue where they reside in the synaptic layers of the retina as well as in the avascular photoreceptor outer segment layer. We also found a number of CD8 T cells residing outside the vessels in the choroid, where they were in close proximity to RPE cells. Following contraction of the virus-specific CD8 T cell population, only few cells remained within the retina. A hallmark of cells found both within and outside vessels in the neural retina and RPE/c was expression of CXCR6, while CXCR3 expressing cells were specifically enriched within the retinal tissue. We found that the CXCR6 ligand, CXCL16, was expressed constitutively in the membrane of RPE cells and that this chemokine was secreted primarily from the apical side of the cells in response to inflammatory stimulation.

It has previously been shown that CXCR6 expression is important for T cell migration and localization within the lungs and liver after infection, and that CXCL16 expression by epithelial cells directs this migration (13–15). The results shown here suggest that similar mechanisms are at play in the retina in spite of immune privilege. RPE cells appear to be at the center of the regulation of CD8 T cell localization within the tissue by expression of CXCL16. CXCL16 can exist in both a membrane bound and a soluble form (24), and RPE cells seem to express both forms. Secretion only occurs when the cells are stimulated with a pro-inflammatory cytokine such as IFN-γ. Apical secretion of chemokines from human RPE cells in response to inflammatory stimulation *in vitro* has previously been shown (16), and here we elaborate on this finding and show that RPE cells behave like epithelial cells in the lungs and gut associated lymphoid tissue (GALT) in that they constitutively express CXCL16 in their membrane (13, 25). Soluble CXCL16 acts as a chemotactic agent for CD8 T cells and the secretion of CXCL16 by RPE cells is therefore likely involved in regulating CD8 T cell migration during inflammatory settings (13–15). The function of membrane bound CXCL16 is not fully characterized, but it has previously been shown to be a phagocytic receptor for bacteria in macrophages (26). However, this does not seem to be the case for CXCL16 expressed by RPE cells, since immunohistochemical staining showed that CXCL16 was expressed on the basolateral side of the plasma membrane rather than on the apical side. In GALT, membrane bound CXCL16 mediates interaction between epithelium and CXCR6^+^ CD8 T cells, and it is plausible that it has the same function in RPE cells (25). Membrane bound CXCL16 could also serve a homeostatic function in RPE cells that has yet to be characterized.

RPE cells are vital for the function of the photoreceptor outer segments as they are the sole providers of nutrients while also being responsible for waste disposing, i.e. they ensure homeostasis and proper function of the retina. At the same time, they constitute what is known as the outer blood retinal barrier since they strictly control access to the photoreceptor layer. For these reasons, it was originally believed that these cells do not contribute to the local immune response in the same manner as other epithelial cells do. However, the results shown here, in combination with previous reports suggest that these cells are in fact immunologically active just like epithelial cell in other organs (9, 16, 21, 27, 28).

CXCR6 expression was found on CD8 T cells from all analyzed compartments and expression increased on infiltrating cells found in the tissue relative to those inside vessels. This was previously reported for lungs, and the authors of that study speculated that this was caused by an antigen encounter. However, no viral antigen could be detected in the retinas of infected animals in the current study, suggesting that while upregulation of CXCR6 upon entry into tissue appears to be a general mechanism, it is not necessarily driven by viral antigen. CXCR3 was also found on CD8 T cells from all analyzed compartments, but expression was specifically enriched in the subset of CD8 T cells found within the retinal tissue. This is in line with previous data from CNS where CXCR3 was important for accumulation of CD8 T cells within the parenchyma (29–31). Further, CXCR3 expression was also up-regulated on tissue resident CD8 T cells within the liver and lung (13, 14). Thus, differential CXCR3 expression on CD8 T cells within the retinal tissue is further indicative of similar mechanisms for immune surveillance of organs in the body irrespective of immunological status and suggests that both CXCR6 and CXCR3 expression are important for migration and positioning within the retina as well as other organs.

In the current study, we used a virus that causes an acute and quickly resolved infection. We did not detect any signs of virus in the retina and no structural changes in the retinal tissue. Contrary to a previous study investigating the impact of CMV infection on the retina (4), only few CD8 T cells remained in the retinal tissue in the long-term. We ascribe this to the inherent differences between the viral infections used. CMV causes a chronic infection associated with a latent state, while LCMV Arm causes only a transient infection. While CMV caused sustained inflammation within the retinal tissue, infection with LCMV Arm caused neither infection nor obvious inflammation of the retina. Combined, our study and the study by Voigt et al. (4) show that CD8 T cells can enter the retina during systemic infection, but in the absence of local virus infection, the majority of the CD8 T cells will disappear during the contraction phase.

In summary, our results show that CD8 T cells gain access to the immune privileged retina as a result of an acute systemic infection. We show that CD8 T cell surveillance of the retina appears to be driven by similar mechanisms to those described for non-privileged tissues such as the lungs and the liver. The study adds to the growing body of evidence that immune privilege may be a more fluid concept than previously believed, and that privilege may be primarily regulated by anti-inflammatory mechanisms within the tissue rather than by physical barriers preventing the entry of immune cells.

## Data availability statement

The original contributions presented in the study are included in the article/**Supplementary Material**. Further inquiries can be directed to the corresponding author.

## Ethics statement

The animal study was approved by The Danish Animal Experiments Inspectorate. The study was conducted in accordance with the local legislation and institutional requirements.

## Author contributions

JC, AT, MN and MS contributed to conception design of the study. EP and MS performed the experiments and analyzed the data. JC, AT, MN, TS, BH, HV and MS contributed to interpretation of the data. MC and HX isolated and characterized the primary RPE cell line. EP and MS wrote the first draft of the manuscript. All authors contributed to manuscript revision, read, and approved the submitted version.

## Supplementary material

Supplementary Figure 1 Viral RNA and GAPDH RNA Ct values and standard curve. **(A)** Ct values for GAPDH in RPE/c, retina and spleen. **(B)** Ct values for viral RNA in spleen. Viral RNA could not be detected in RPE/c and retina. **(C)** Standard curve for amplification of LCMV NP from viral RNA purified from the virus stock used for infection.

Supplementary Figure 2 Gating strategy for flowcytometry. Gating strategy shown for neuroretina from an infected animal (d. 8).
