# Supplementary figures and images for: Systemic virus infection results in CD8 T cell recruitment to the retina in the absence of local virus infection
Source: Front Immunol. 2023 Aug 18;14:1221511. doi: 10.3389/fimmu.2023.1221511 (PMC10471971; doi:10.3389/fimmu.2023.1221511)

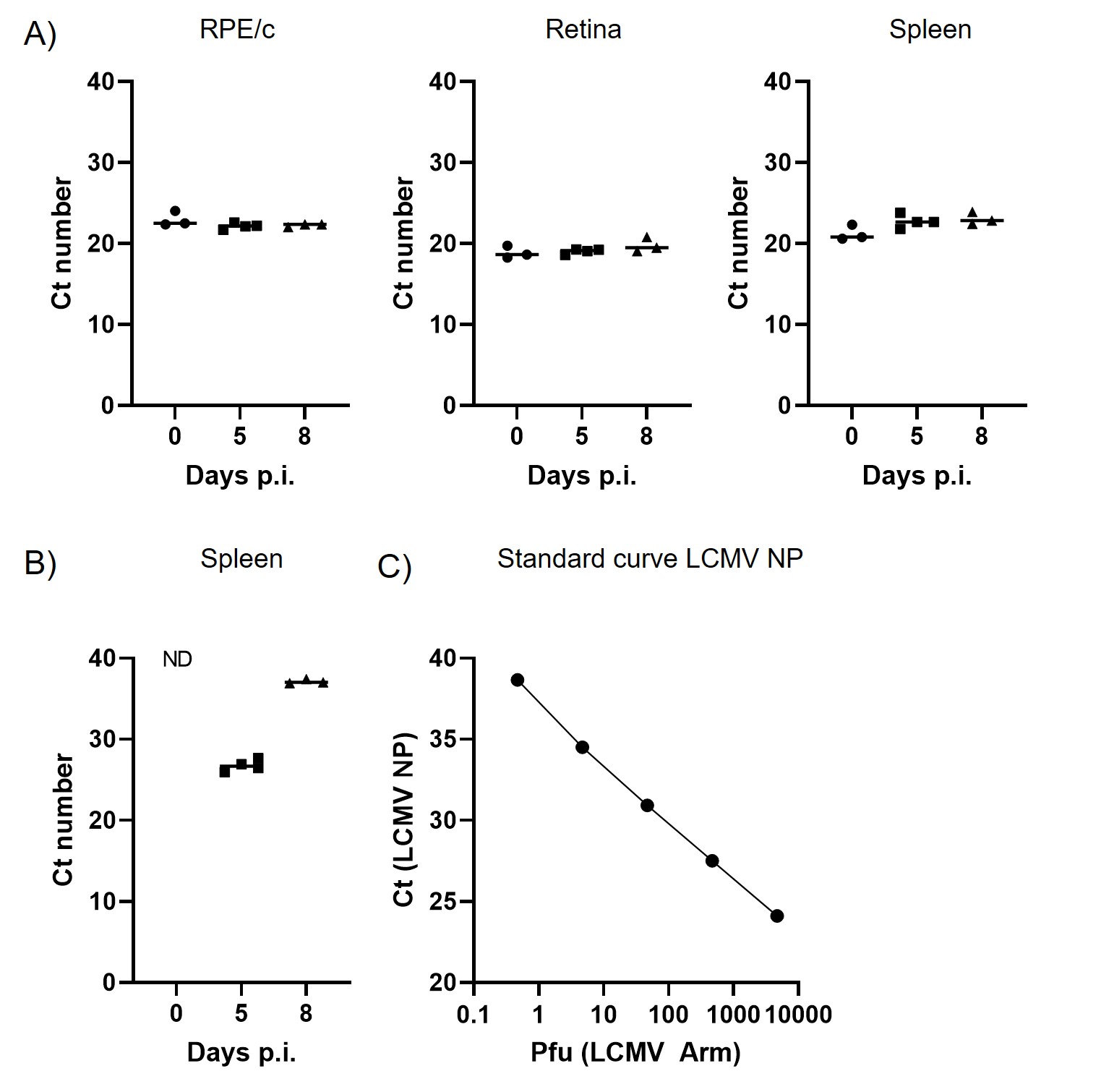

Supplement: Supplementary Figure 1 — Viral RNA and GAPDH RNA Ct values and standard curve. (A) Ct values for GAPDH in RPE/c, retina and spleen. (B) Ct values for viral RNA in spleen. Viral RNA could not be detected in RPE/c and retina. (C) Standard curve for amplification of LCMV NP from viral RNA purified from the virus stock used for infection. [file Image_1.jpg]

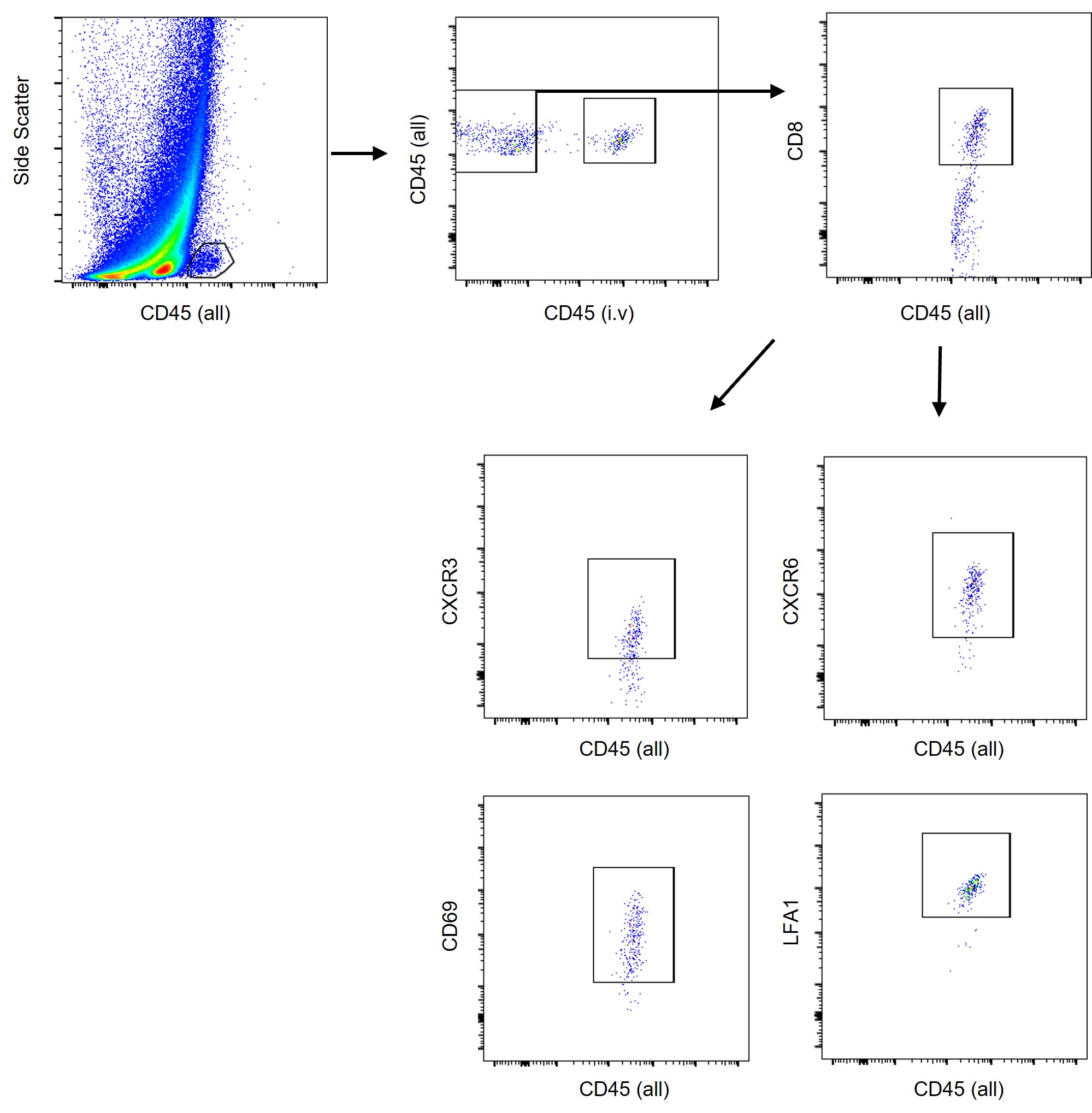

Supplement: Supplementary Figure 2 — Gating strategy for flowcytometry. Gating strategy shown for neuroretina from an infected animal (d. 8). [file Image_2.jpeg]
